# Supplementary material for: Common eating habit patterns are associated with a high maximum occlusal force and pre-eating cardiac vagal tone
Source: PeerJ. 2023 Mar 23;11:e15091. doi: 10.7717/peerj.15091 (PMC10040181; doi:10.7717/peerj.15091)
Supplement: Supplemental Information 1 — Table source: Okada M, Okada K, Kakehashi M (2022) Eating habit patterns may predict maximum occlusal force: A preliminary study. PLoS ONE 17(2): e0263647. https://doi.org/10.1371/journal.pone.0263647; https://doi.org/10.1371/journal.pone.0263647.s001. [file peerj-11-15091-s001.pdf]

Eating Habit Questionnaire

Eating habit item	Question	Answers
1. Breakfast habits	Are you in the habit of eating breakfast?	☐ Skip sometimes ☐ Every day
2. Always eat at a fixed time	Do you usually eat at fixed times?	☐ Yes ☐ No
3. Number of meals per day (including snacks)	How many meals do you eat a day (including snacks)?	☐ meals/day
4. Amount eaten	How much food do you usually eat?	☐ Small ☐ Medium ☐ Large
5. Eating speed	How quickly do you eat?	☐ Slow ☐ Fast
6. Chew food well	Do you chew your food well?	☐ Yes ☐ No
7. Eat until full	Do you eat until you are full?	☐ Yes ☐ No
8. Think about the nutritional balance of the meal	Do you pay attention to the nutritional balance of your meal?	☐ Yes ☐ No
9. Many likes and dislikes	Do you have many food likes and dislikes?	☐ Yes ☐ No
10. Eat for stress relief	Does eating relieve your stress?	☐ Yes ☐ No
11. Eat with others or alone (including family)	Are you always with someone when you eat (including family)?	☐ Alone ☐ Sometimes eat with others ☐ Always eat with others
12. Conversation when eating	Do you have a conversation when eating?	☐ No conversation ☐ Sometimes conversation ☐ Always conversation
