# Supplementary material for: Common eating habit patterns are associated with a high maximum occlusal force and pre-eating cardiac vagal tone
Source: PeerJ. 2023 Mar 23;11:e15091. doi: 10.7717/peerj.15091 (PMC10040181; doi:10.7717/peerj.15091)
Supplement: Supplemental Information 2 [file peerj-11-15091-s002.pdf]

食事習慣質問票

食事習慣項目	質問	回答
1. 朝食習慣	朝食を食べる習慣はありますか？	☐ 時々食べない ☐ いつも食べる
2. 食事をする時間	あなたはいつも決まった時間に食事をしますか？	☐ はい ☐ いいえ
3. 食事回数（間食を含む）	一日何回食事をしますか（間食を含む）？	☐ 回/一日
4. 食事の量	あなたの食事量はどれくらいですか?	☐ 少ない ☐ 中程度 ☐ 多い
5. 食事の速さ	食べる速さはどれくらいですか?	☐ 遅い ☐ 速い
6. 咀嚼の度合い	あなたは、食べ物をよく噛んで食べますか？	☐ はい ☐ いいえ
7. 食事における満腹度合い	あなたは、お腹いっぱいになるまでたべますか？	☐ はい ☐ いいえ
8. 食事の栄養バランス思考	食事の栄養バランスに気をつけますか？	☐ はい ☐ いいえ
9. 食べ物の好き嫌い	食べ物の好き嫌いは多いですか？	☐ はい ☐ いいえ
10. 食事でストレス解消	食べることでストレスが解消しますか？	☐ はい ☐ いいえ
11. 集団で食事及び孤食 (家族を含む)	食事をするときは、いつも誰かと一緒に食べますか (家族を含む)？	☐ 一人でたべる ☐ 時々誰かと食べる ☐ いつも誰かと食べる
12. 食事時の会話	食事中に会話をしますか？	☐ 会話をしない ☐ 時々会話をする ☐ いつも会話をする
