# Supplementary figures and images for: Common eating habit patterns are associated with a high maximum occlusal force and pre-eating cardiac vagal tone
Source: PeerJ. 2023 Mar 23;11:e15091. doi: 10.7717/peerj.15091 (PMC10040181; doi:10.7717/peerj.15091)

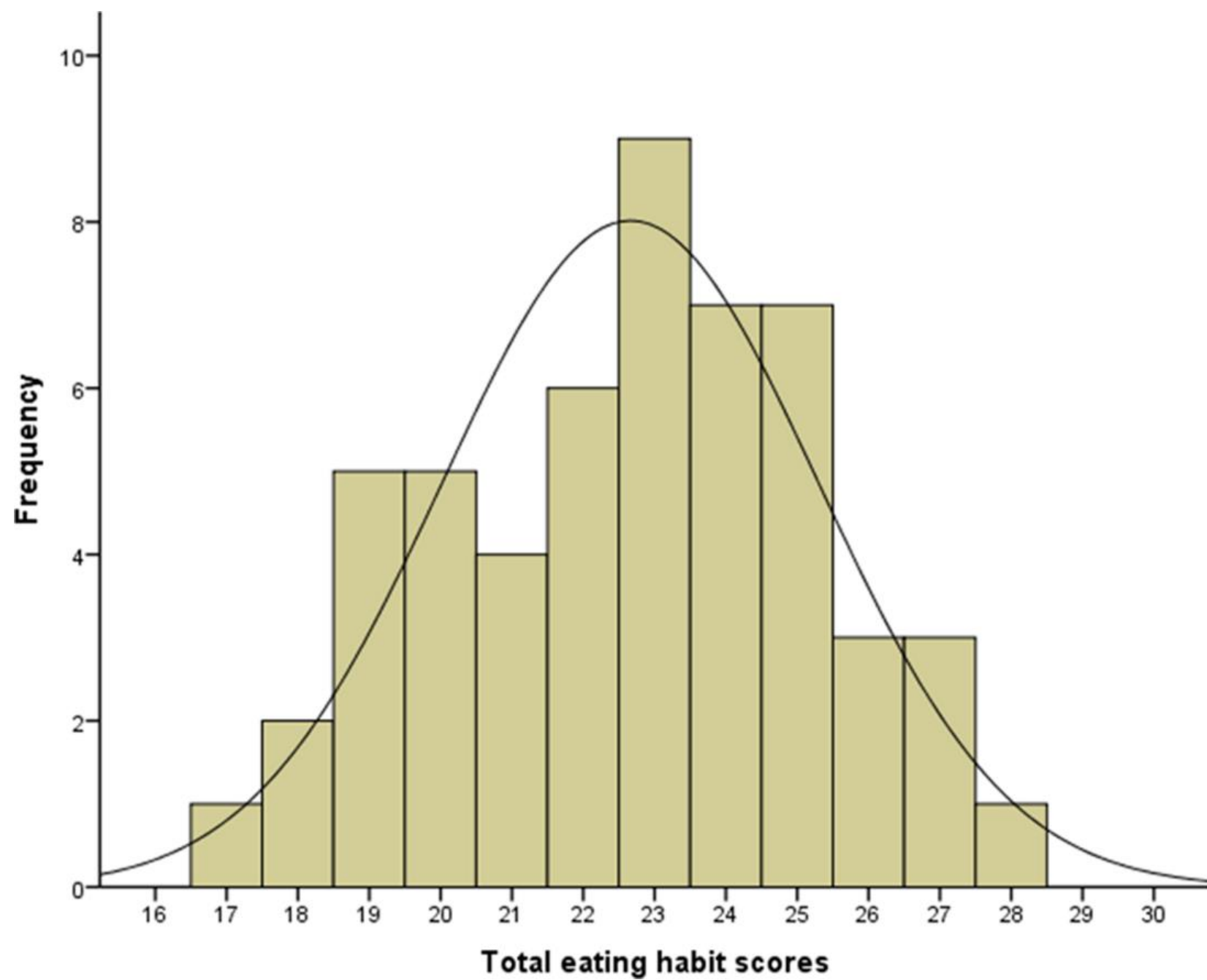

Supplement: Supplemental Information 3 — Figure source: Okada M, Okada K, Kakehashi M (2022) Eating habit patterns may predict maximum occlusal force: A preliminary study. PLoS ONE 17(2): e0263647. https://doi.org/10.1371/journal.pone.0263647; https://doi.org/10.1371/journal.pone.0263647.g001 [file peerj-11-15091-s003.pdf]

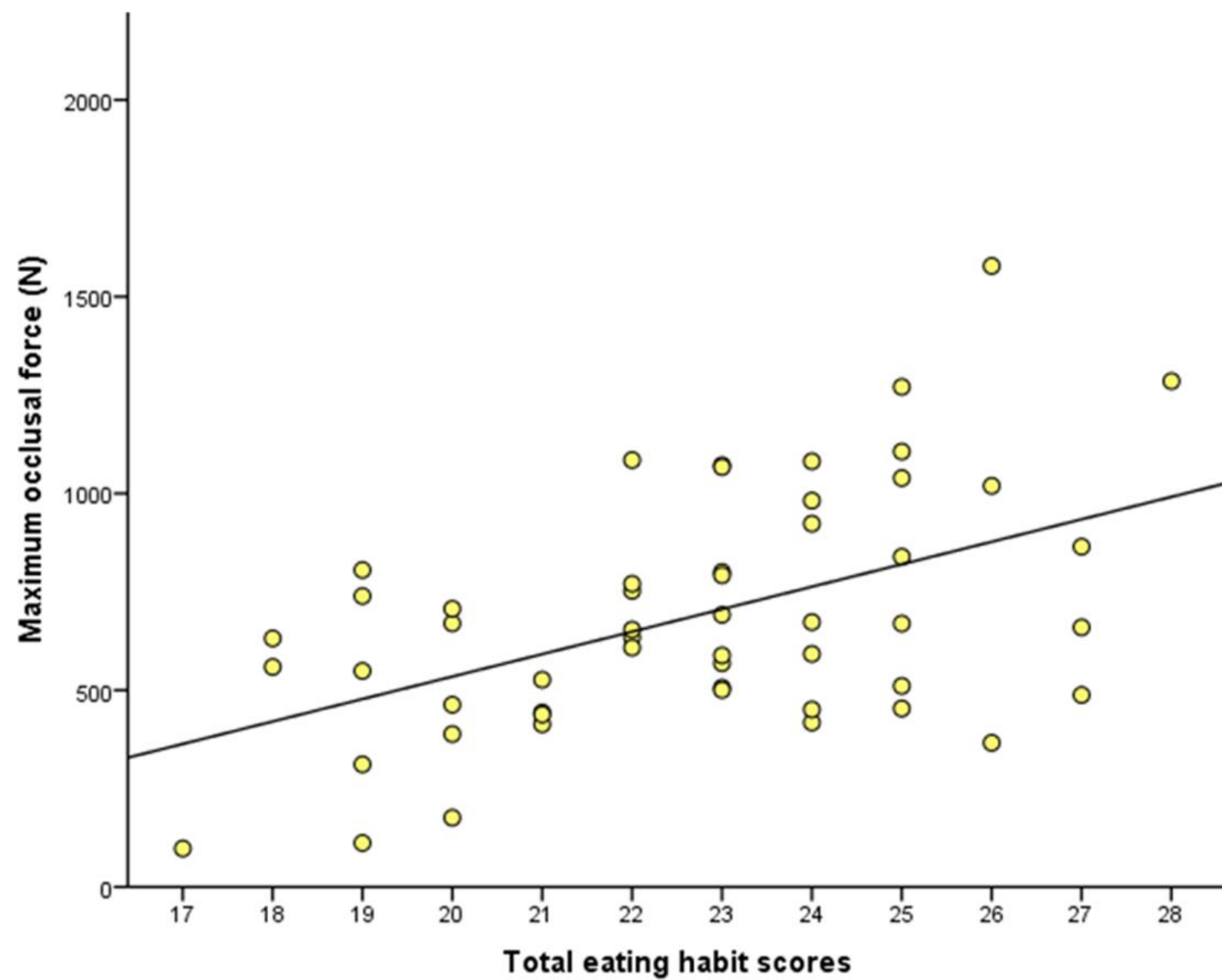

Supplement: Supplemental Information 4 — Figure source: Okada M, Okada K, Kakehashi M (2022) Eating habit patterns may predict maximum occlusal force: A preliminary study. PLoS ONE 17(2): e0263647. https://doi.org/10.1371/journal.pone.0263647; https://doi.org/10.1371/journal.pone.0263647.g002. [file peerj-11-15091-s004.pdf]

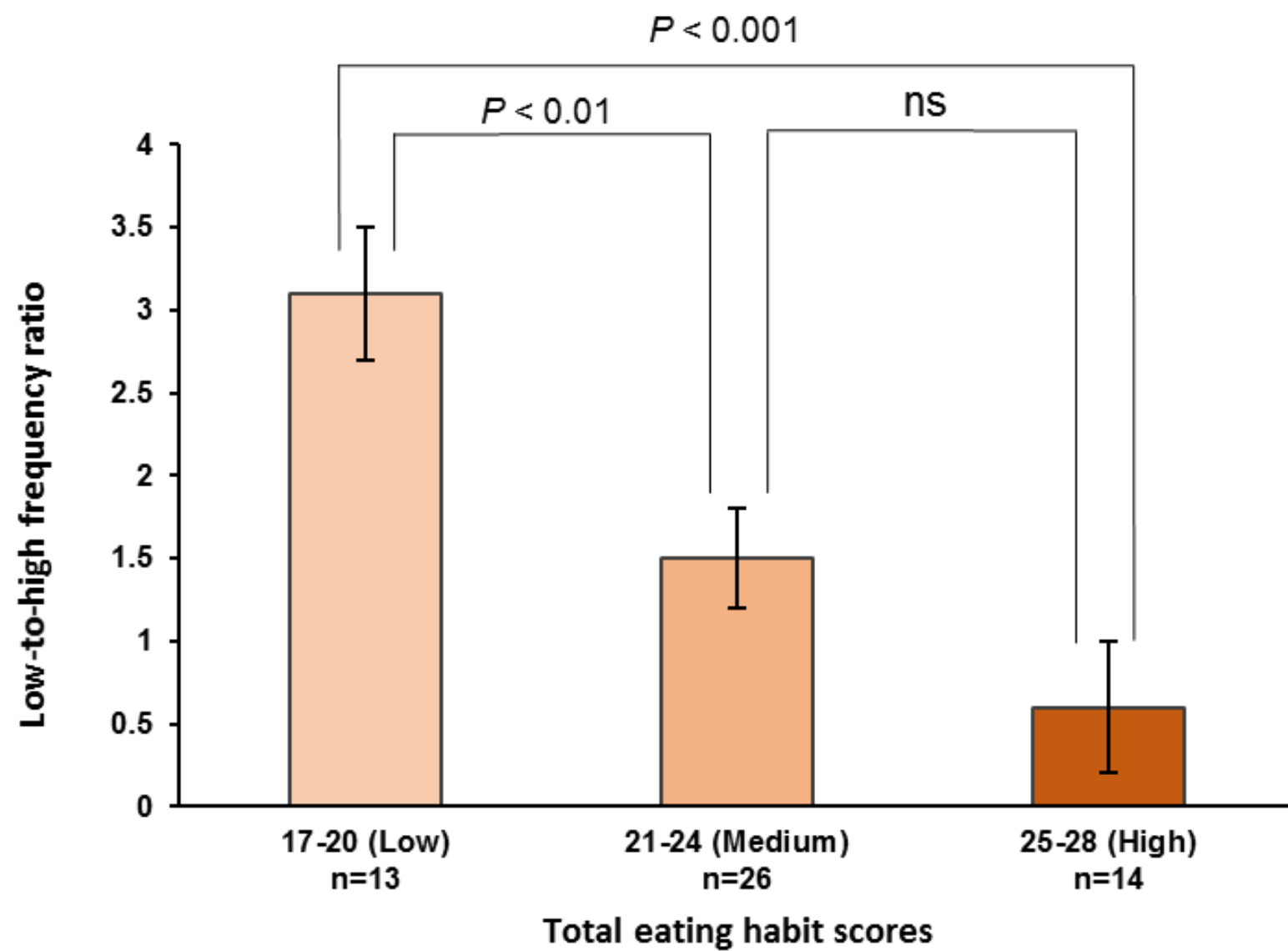

Supplement: Supplemental Information 5 [file peerj-11-15091-s005.pdf]
